# Development and Reliability of a Clinician-rated Instrument to Evaluate Function in Individuals with Shoulder Pain: A Preliminary Study

**DOI:** 10.1002/pri.1555

**Published:** 2013-05-28

**Authors:** Yngve Rol, Benjamin Haldorsen, Ida Svege, Astrid Bergland

**Affiliations:** 1 Faculty of Health Sciences, Oslo and Akershus University College of Applied SciencesOslo, Norway; 2 Department of Physiotherapy, Martina Hansen's HospitalBærum, Norway; 3 Norwegian research center for Active Rehabilitation (NAR)Oslo, Norway

## Abstract

**Background and Purpose:**

Subacromial impingement syndrome (SIS) is a common and disabling condition in the population. Interventions are often evaluated with patient-rated outcome measures. The purpose of this study was to develop a simple clinician-rated measure to detect difficulties in the execution of movement-related tasks among patients with subacromial impingement syndrome.

**Method:**

The steps in the scale development included a review of the clinical literature of shoulder pain to identify condition-specific questionnaires, pilot testing, clinical testing and scale construction. Twenty-one eligible items from thirteen questionnaires were extracted and included in a pilot test. All items were scored on a five-point ordinal scale ranging from 1 (no difficulty) to 5 (cannot perform). Fourteen items were excluded after pilot testing because of difficulties in standardization or other practical considerations. The remaining seven items were included in a clinical test-retest study with outpatients at a hospital. Of these, four were excluded because of psychometric reasons. From the remaining three items, a measure named Shoulder Activity Scale (summed score ranging from 3 to 15) was developed.

**Results:**

A total of 33 men and 30 women were included in the clinical study; age range 27–80 years. The intraclass correlation coefficient results for inter-rater reliability and test-retest reliability were 0.80 (95% CI = 0.51–0.90) and 0.74 (95% CI = 0.58–0.84), respectively. The standard error of measurement and minimal detectable change were 1.19 and 3.32, respectively. The scale was linked to the International Classification of Functioning, Disability and Health second level categories lifting and carrying objects (d430), dressing (d540), hand and arm use (d445) and control of voluntary movement (b760).

**Conclusion:**

The Shoulder Activity Scale showed acceptable reliability in a sample of outpatients at a hospital, rated by clinicians experienced in shoulder rehabilitation. The validity of the scale should be investigated in future studies before application to common practice. © 2013 The Authors. Physiotherapy Research International published by John Wiley & Sons Ltd.

## Introduction

Shoulder pain is an umbrella term for conditions with different aetiologies and courses, and prevalence estimates have varied between 7% and 26% (Luime *et al*., 2004, van der Heijden, 1999). Subacromial impingement syndrome (SIS) is probably the most common shoulder diagnosis, and the condition is associated with substantial loss of function (Neumann, 2010, Silva *et al*., 2008, Lewis *et al*., 2005, van der Windt *et al*., 1995). SIS is describing a dysfunctional mechanism, and the alterations in movement patterns associated with the condition have been extensively analysed (Bigliani and Levine, 1997, Michener *et al*., 2003, Neumann, 2010, Lin *et al*., 2006, Ludewig and Cook, 2000, Lukasiewicz *et al*., 1999). It is essential that the alterations in movement patterns are also included in functional assessments in the clinic, but few such standardized measures are available.

Reliable and valid standardized measures are important for clinical decision making and research. Patient-rated outcome measures have been recommended to evaluate interventions in patients with shoulder pain, and a number of condition-specific measures are now available (Bot *et al*., 2004, Michener, 2011). Clinician-rated methods are also considered important in assessments, but the most commonly used measures are either a standardization of the clinical examination or physical examination tests (Constant and Murley, 1987, Richards *et al*., 1994, Hegedus *et al*., 2008). Although the patient-rated and clinician-rated condition-specific measures probably capture different aspects of functioning, few efforts have been made to analyse the content.

The International Classification Of Functioning, Disability and Health (ICF), provides a framework for describing and classifying the content of all measures of function (WHO, 2001). The ICF is based on an integrative model covering functioning within its components of *body functions (b)*, *body structures (s)*, *activities and participation (d)* and the *environmental (e)* and *personal factors* (not classified). The ICF classification provides categories of functioning and environmental factors that are arranged in a hierarchical fashion by using an alphanumeric coding system; the first letter referring to the component, followed by a numeric code that starts with the chapter number (e.g. *mobility*, d4-chapter), followed by the second level (e.g. *hand and arm use*, d445), third level (e.g. *reaching*, d4452) and fourth level when appropriate. Because of a generic structure, the categories at a lower level are included in the higher level categories and chapters. Procedures have been established to classify the content of functional measures by ICF categories, regardless of their purpose, extent and by whom they are rated (Cieza *et al*., 2002, Cieza *et al*., 2005).

According to the ICF, the traditional clinician-rated measures may be referred to as belonging to the body functions and structures components, whereas the available patient-rated questionnaires to the activities and participation (Michener, 2011). To our knowledge, no clinician-rated measure containing content relating to the activities & participation component of the ICF has been developed. The clinician-rated measures have the advantage of directly measuring the unit of interest; they reflect the current situation and are less vulnerable to the patient's recall, language and problems with vision or literacy (Gotay, 1996). Patient and clinician ratings probably reflect different constructs, and a low to moderate correlation has been reported (Reneman *et al*., 2002, Mannerkorpi *et al*., 2006, Stratford and Kennedy, 2006). The aim of this study was to develop a reliable clinician-rated functional scale to measure change over time, according to the ICF component activities and participation, in patients with SIS.

## Methods

### Scale development

**Figure 1:**
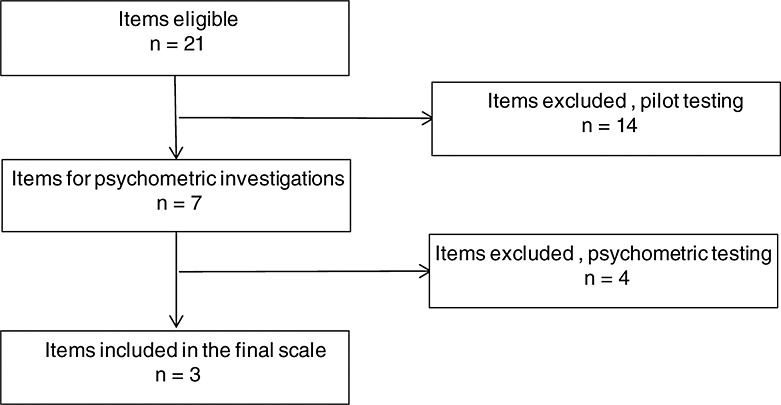
Flowchart of the item reduction process

The steps in the scale development included a review of the scientific literature of shoulder pain, pilot testing, clinical testing and scale construction (Clark and Watson, 1995, Loevinger, 1957, Streiner and Norman, 2008) (Figure 1). Thirteen frequently used condition-specific questionnaires of shoulder function were identified after a review of the scientific literature. From these, 21 single items were extracted and considered eligible for pilot testing after discussions between the researchers (YR, BH and IS). All items described the execution of tasks with dynamic movements of the arm at or above shoulder level. With the participation of outpatients with shoulder pain at a hospital, the eligible items were further investigated in a pilot test. The researchers (BH and IS) and other experienced physiotherapists at the hospital participated as observers. As a result of the pilot test, 14 items that were difficult to standardize or gave little information about the patient's movement patterns were excluded. Decisions were based on agreement between all observers. In cases of disagreement, a senior member of the research group (AB) was consulted. There were no examples of such disagreement. The remaining 7 items were included in a full-scale clinical study for investigation of reliability and representation in the ICF classification.

To rate the magnitude of a functional problem, a five-point ordinal scale similar to the qualifiers in the ICF classification was used (WHO, 2001). The anchor points of the scale were no difficulty (1), mild difficulty (2), moderate difficulty (3), severe difficulty (4) and cannot perform (5). No definition of the term difficulty was given, as it was assumed that physical therapists experienced in shoulder rehabilitation have a common understanding of the term. The intervals between the categories were not further investigated but treated as equal in the statistical analyses.

All items were linked to second level ICF categories according to established rules (Cieza *et al*., 2005). Inter-item and item-to-sum correlations and representation in the ICF classification were used as exclusion criteria. A tentative summed scale named Shoulder Activity Scale (SAS) was constructed from the remaining three items and further statistically examined Appendix 1). The included items were *lifting an object to a shelf*, *putting on a jacket* and *moving an arm sideways*. All items were weighted equal, and the scale had a possible range of 3 (no difficulties) to 15 (cannot perform). The scale was easy to administer and was in most cases completed within 5 minutes. No adverse effects from performing the SAS items were reported by the subjects or identified by the raters.

The items were linked to the ICF second level categories *lifting and carrying objects* (d430), *dressing* (d540) and *hand and arm use* (d445), respectively. The aim of the scale, to measure difficulty in terms of altered movement patterns, was linked to the *control of voluntary movement* (b760) category.

### Subjects

A clinical test-retest study with outpatients attending the orthopaedic division at a hospital between December 2007 and October 2010 was conducted. The eligible patients were non-native English speakers. Inclusion criteria were primary diagnosis of SIS according to standardized criteria (Juel *et al*., 2008, Walker-Bone *et al*., 2003). Exclusion criteria were systematic inflammatory disease or generalized pain, cardiac disease, symptoms of cervical spine disease or surgery in the affected shoulder within the last 6 months.

### Power analysis

A method for sample size based on the intraclass correlation coefficient (ICC), was chosen (Walter *et al*., 1998). The minimally acceptable ICC value (*ρ*_1_ = 0.7) versus an alternative ICC value reflecting the expectations (*ρ*_1_ = 0.8) was chosen. With a power of 80% (*β* = 0.2) and a significance level of 5%, a sample size of at least 40 patients was required (Walter *et al*., 1998).

### Procedure and measures

Descriptive information was collected for all participants. The items were tested twice for each participant without any treatment in between. The instruction to the patients was as far as possible provided in a standardizxed manner and is shown in Appendix 1. The average time between baseline test and retest was 7.5 days (range 7–21). The participants were asked on the day of retest whether a substantial change in their shoulder condition had occurred since the baseline test. Participants were included in the further analyses regardless of whether a substantial change in their condition had occurred. Two independent clinicians took part in the testing at baseline, where one participated at retest. A total of five clinicians participated in the test sessions; all experienced in shoulder rehabilitation at the hospital. All clinicians had participated in a standardized training session before conducting the test sessions.

Participants also completed the Shoulder Pain and Disability Index (SPADI) at baseline test (Roach *et al*., 1991). SPADI is a patient-rated measure for patients with shoulder pain consisting of 13 questions, divided in the domains pain (5 items) and disability (8 items). Each item is rated on a numerical scale from 0 (best) to 10 (worst) and summed up to a domain score. Each domain score is equally weighted then added for a total percentage score ranging from 0 to 100.

### Statistical analysis

The statistical analysis was conducted with the ibm spss Statistics 19 for windows (IBM Corporation, New York, USA) and the stata/IC 11.1 for Mac (StataCorp LP, Lakeway Drive, Texas, USA).

The mean values or frequencies with the standard deviations (SD) were reported for the numerical or categorical variables. The association between the SAS scores and age and duration of symptoms was investigated with estimations with Pearson's product–moment correlation coefficient (*r*) and visual inspection of bivariate data for non-linear relations.

For further investigation of reliability, the following underlying measurement properties were chosen (Mokkink *et al*., 2010, Terwee *et al*., 2007): internal consistency, reliability and measurement error. Internal consistency was estimated with Cronbach's alpha. An alpha between 0.7 and 0.9 was considered fair. Consistency and unidimensionality was further investigated with inter-item correlations estimated with Pearson's product–moment correlation coefficient (Cortina, 1993). Inter-item correlations in the range of 0.15–0.50 and mean inter-item correlations of 0.40–0.50 were considered acceptable (Clark and Watson, 1995). Inter-rater reliability and test-retest reliability was estimated with the ICC. To be able to generalize the results to a population of other clinicians and because the difficulty of the items was considered to be a systematic source of variance, a two-way random effect model single measure reliability had to be chosen (Shrout and Fleiss, 1979, McGraw and Wong, 1996).

The measurement error was defined as the systematic and random error of a patient's score that was not attributed to true changes in the construct to be measured (Mokkink *et al*., 2010). The standard error of measurement (SEM), which reflects the standard deviation of the distribution of the patient's score, with no change in health status and no learning effect taking place, was used (Wyrwich, 2004, Weir, 2005). There are two types of SEM: *SEM*_agreement_ and *SEM*_consistency_. To take the systematic difference into account, the *SEM*_agreement_ was chosen, estimated with the formula 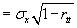, where (σ*_x_*) was the pooled standard deviation of test and retest scores, and (*r_tt_*) was the reliability coefficient. From the SEM value, it is possible to estimate the minimal detectable change (MDC), which is the smallest change that can be defined by the instrument beyond measurement error (de Vet *et al*., 2006, Beckerman *et al*., 2001). The following formula was used: 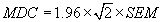, where 2 relates to test and retest, and 1.96 relates to the 95% confidence interval. A plot with the difference of the baseline and retest versus the mean of the sum scores was drawn (Bland and Altman, 1999). The limits of agreement (LOA) were plotted as the standard deviation of the mean difference (SD) multiplied by ±1.96.

All the participants signed a written consent, and the study was approved by the Norwegian Regional Committee for Ethics and conducted according to the Helsinki Declarations.

## Results

Sixty-three patients, thirty women and thirty-three men participated in the clinical study. Ninety-four met the inclusion criteria, twenty-nine did not accept participation, two were excluded because of generalized pain and three dropped out between baseline test and retest. No descriptive data were recorded on eligible patients who did not accept participation. The mean age of the participants was 53.3 years (SD = 12.9). The mean duration of symptoms was 46.6 months (SD = 72.3). Thirty-eight of the participants were working, eight were sick listed and seventeen were retired, receiving disability benefit or unemployed. There were 30 cases of pain in the right shoulder, 19 in the left shoulder and 14 cases of bilateral pain. The dominant arm was affected in 30 of the cases. Five patients reported a substantial change of the condition during the test period. The mean SPADI score at baseline was 36.2 (SD = 16.6).

The item-to-item correlations ranged between 0.30 and 0.49, and the item-to-total between 0.70 and 0.82 (Table 1). The Cronbach's alpha of consistency for the SAS sum score was estimated at α = 0.86. There were no significant correlations or non-linear associations between the participants' ages or permanence of symptoms and the SAS score.

**Table 1:** Significant inter-item and item-to-sum correlations with Person's *r* in the baseline test scores (*n* = 63)

Item	Putting on a jacket	Moving an arm sideways	Shoulder Activity Scale sum score
Lifting an object to a shelf	0.30	0.49	0.77
Putting on a jacket		0.34	0.70
Moving an arm sideways			0.82

**Figure 2:**
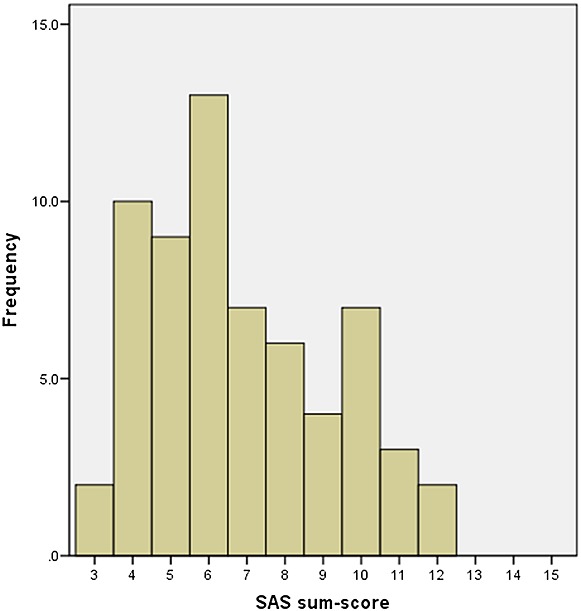
Histogram with the distribution of Shoulder Activity Scale sum scores at the baseline test (*n* = 63)

The distribution of the scale were positively skewed as two participants had an SAS score of 3 and none above 12 (Figure 2).

The moving the arm sideways had a higher mean score than the other items, indicating that it was a more difficult task (Table 2).

**Table 2:** Reliability estimates (*n* = 60) with pooled test-retest mean, range and inter-rater reliability, test-retest reliability, standard error of measurement (SEM), minimal detectable change (MDC) and effect size for single items (1–5) and sum score (3–15)

Item	Mean (SD)	Range	ICC inter-rater (95% CI)	ICC test-retest (95% CI)	SEM	MDC
Lifting an object to a shelf	1.87 (0.98)	1–5	0.66 (0.35–0.82)	0.59 (0.40–0.73)	0.61	1.69
Putting on a jacket	1.94 (0.98)	1–5	0.71 (0.42–0.85)	0.55 (0.35–0.71)	0.62	1.72
Moving an arm sideways	3.00 (1.15)	1–5	0.75 (0.61–0.84)	0.84 (0.75–0.90)	0.45	1.25
SAS sum score	6.81 (2.38)	l3–12	0.80 (0.51–0.90)	0.74 (0.58–0.84)	1.19	3.30

**Figure 3:**
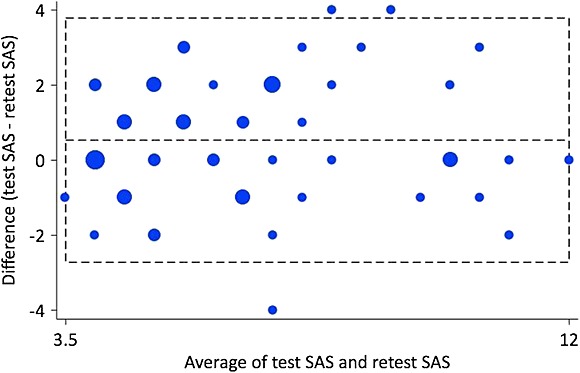
Intra-individual differences (*n* = 60) plotted against the difference between test and retest scores on Shoulder Activity Scale. The central horizontal line represents the mean difference, whereas the flanking lines represent the 95% limits of agreement

The difference between SAS test and retest was plotted against the average, with the 95% limits of agreement at −2.72 and 3.79 (Figure 3). The mean difference was 0.53. Three out of sixty values were outside the LOA.

## Discussion

The aim of this study was to develop a reliable clinician-rated functional scale to measure change over time, according to the ICF component activities and participation, in patients with SIS.

The main results of the clinical study were the findings of an inter-rater reliability and test-retest reliability of the SAS of 0.80 and 0.74, respectively (Table 2), in line with what was expected in the power analysis. There is no commonly agreed limit for what should be considered an acceptable ICC value, but an ICC above 0.70 with the lower limit of the confidence interval above 0.60 has been proposed in clinician-rated methods (Terwee *et al*., 2006). Even though both reliability estimates exceeded the minimum recommendations, the lower limits of the 95% confidence interval for both estimates were slightly below 0.60. The acceptable reliability found in the current study were in line with previous findings of Westerberg and colleges who concluded that three active motor tests had good reliability when used as functional tests in painful shoulders (Westerberg *et al*., 1996).

The inter-item correlations (Table 1) in the final scale was within what was considered acceptable, ranging from 0.30 to 0.49 (Clark and Watson, 1995). An internal consistency of 0.88 indicates that no items were redundant or measured other constructs. Other possible combinations of items resulted in lower alpha values. The three items were most likely not equally difficult as the item moving an arm sideways had a higher mean score (Table 2). However, the item had an acceptable inter-item correlation and item-to-total correlation (Table 1). The problems of different item-difficulty in scales are shared with other scales developed through statistical analysis based on classical test theory.

The MDC for the SAS was estimated to 3.30 (Table 2). The interpretation is that individual changes in the sum score of 1–3 points can be due to systematic or random errors. In classical test theory, the MDC is considered a stable property of the instrument, and a change in the sum score of 4 or higher should thus be considered real but not necessarily clinically relevant (de Vet *et al*., 2006). The MDC should not be interchanged with the minimal important difference, which refers to the benefit of treatment in a specific population (de Vet *et al*., 2006, de Vet and Terwee, 2010). Controversy exists whether the benefit of treatment estimates should be derived from distribution-based or anchor-based methods. Norman and colleagues found consistent evidence that the minimal important difference equals close to half of an SD at baseline in a systematic literature review where both anchor-based and distribution-based methods had been used (Norman *et al*., 2003). Furthermore, Wyrwich suggested a one-to-one relation between the minimal important difference and the SEM (Wyrwich, 2004). Estimates based on the aforementioned distribution-based methods resulted in a minimal important difference of 1.19 in both cases. According to the estimation methods recommended by Norman and Wyrwich, an SAS sum score of at least 4 is also clinically important.

The participants had a high functional level measured with SPADI, compared with other studies including patients with subacromial conditions (Ekeberg *et al*., 2008, Williams *et al*., 1995). There were only two patients with the lowest SAS score of 3, and none with the sum scores 13–15 (Figure 2). Even though the distribution was obviously skewed, this is less than the 15% normally considered a floor effect (Terwee *et al*., 2007). A skewed distribution however should not necessarily be considered a problem in functional scales but rather a common and logical manifestation of the underlying construct (Streiner and Norman, 2008). The LOA-plot (Figure 3) gives a graphical expression of the ability of an instrument to replicate observations, and the differences should ideally be close to zero (Bland and Altman, 1999). The plot gives a visual indication of a slightly higher retest score among most participants, consistent for both low and high SAS average scores.

The items in SAS were linked to ICF categories from the *mobility* (d4-chapter) or *self-care* (d5-chapter) of the *activities and participation* component, and the aim of the scale was linked to the *neuromusculoskeletal and movement-related functions* (b7-chapter) of the *body functions* component (WHO, 2001). To our knowledge, no other similar clinician-rated activity scale exists. The standardized clinical examination methods and the physical examination tests commonly used in the assessments have no content relating to the *activities and participation* component of the ICF (Constant and Murley, 1987, Hegedus *et al*., 2008, Richards *et al*., 1994). The FiT-HaNSA-test focuses on muscle endurance, which is also covered by the *body functions* component (MacDermid *et al*., 2007). Hence, the test probably measures a different construct than the SAS.

The SAS needs to be validated before implemented into clinic. Nevertheless, the current study may contribute to increase the attention on the content of functional assessments in patients with shoulder pain. The study may facilitate a further use of the ICF to classify functional measures. Future work should further investigate how standardized clinician-rated measures may be implemented in functional assessments and how they relate to the patient-rated measures.

### Study limitations

First, the SAS is based on the assumption that clinicians have a common understanding of the term *difficulty*. Although the assumption is supported by the findings of the current study, it may have contributed that all the raters were working at the same hospital. No commonly agreed on guidelines for assessments of shoulder pain yet exists. Second, the treatment of ordinal data as numerical in the statistical analyses may be questioned, because no investigations of the intervals between the anchor points had been conducted. The approach was chosen because of the fact that most statistical methods used in psychometric evaluations require numerical data (Streiner and Norman, 2008). Third, it should be recognized that the test was applied to a non-native English-speaking population, and it is thus possible that native English-speaking patients might interpret the instructions differently.

## Conclusions

The SAS seems to be a reliable clinician-rated instrument to measure functional change in patients with SIS. A change score of at least 4 points is required for evaluation of individual patients. Time of administration was less than 5 minutes, and no specialized equipment is required. The content of the scale is covered by the mobility (d4-chapter) and self-care (d5-chapter) of the ICF. The validity of the scale needs to be established before it is applied to common practice.

## Appendix

**Table 1:** Appendix: Shoulder Activity Scale

			Score (circle the most relevant)				
Test	Procedure	Instruction	No difficulty	mild difficulty	Moderate difficulty	Severe difficulty	Cannot perform
1. Lifting an object to a shelf	From a standing or sitting position, the subject lifts a 1-kg object from a table to a high shelf. The task is repeated three times without a break. The height of the shelf should be slightly above the subject's head, and the difference in height between the table and the shelf is at least 0.7 m.	*Lift the object up on the shelf and back on the table three times*.	1	2	3	4	5
2. Putting on a jacket	From a standing or sitting position, the subject puts on a jacket with the healthy arm in the first sleeve and then off beginning with the painful arm. The jacket should be medium tight and made of non-stretchy material.	*Put on the jacket with the healthy arm in the first sleeve and take it off with the painful arm first*.	1	2	3	4	5
3. Moving an arm sideways	From a sitting position, with approximately 90° angle in the hip and knee, the subject lifts a 2-kg object with a straight and approximately 90° internal rotated arm, from a table in front and to the height of the shoulder. The arm is now at 90° flexion, internal rotated in a sagittal plane. The straight arm is abducted to the frontal plane, and adducted to the sagittal plane without allowing any variation in the height or the rotation of the arm. The task is repeated once without a break.	*Lift the object up from the desk to shoulder height with a straight arm*. *Keep the upper body stable*. *Move the object sideways until the arm is outside the shoulder*, *and then back to forward position*. *Keep the arm at shoulder level and straight through the movement*. *The task is repeated once without a break*.	1	2	3	4	5
Sum-score	1 + 2 + 3 = ____ points						
